# Navigating Separated Instrument Retrieval: A Case Report

**DOI:** 10.7759/cureus.50559

**Published:** 2023-12-15

**Authors:** Manoj Chandak, Paridhi Agrawal, Nikhil Mankar, Swayangprabha Sarangi, Jay Bhopatkar

**Affiliations:** 1 Department of Conservative Dentistry and Endodontics, Sharad Pawar Dental College and Hospital, Datta Meghe Institute of Higher Education and Research, Wardha, IND

**Keywords:** endodontic mishaps, fractured fragment, masseran, broken instrument, instrument retrieval

## Abstract

Instrument separation during endodontic therapy is a complication occurring in 2% to 6% of cases. Focusing on the Masserann technique, the study presents a success rate of 55% in retrieving separated instruments. The technique's effectiveness is demonstrated through a case involving retrieving an instrument from the mandibular first premolar. The technique utilizes various tools, including trephine burs and an extractor, providing a reliable means to dislodge tightly wedged fragments. Despite limitations in specific tooth types and the necessity for frequent radiographic monitoring, the Masserann kit proves effective and underscores the importance of attempting retrieval before considering surgical interventions. The presented case exemplifies the technique's clinical applicability and positive outcomes in intricate scenarios, emphasizing its significance in endodontic practice.

## Introduction

The occurrence of instrument separation during endodontic therapy poses a challenging situation, with reported incidences ranging from 2% to 6% in investigated cases [1]. The presence of a distinct instrument within the root canal obstructs access to the root apex during nonsurgical root canal therapy. These instruments commonly encompass a variety of types, including files, reamers, peeso reamers, Gates-Glidden drills, thermomechanical compactors for gutta-percha compaction, Lentulo spirals, or the tips of specific hand instruments such as gutta-percha spreaders or explorers [2].

Common etiologies of file separation include incorrect use, restrictions in its physical properties, insufficient access, aberrant anatomy of the root canal, and possible manufacturing flaws [2]. If the broken piece protrudes from the root apex, it may irritate the periapex or obstruct comprehensive root canal shaping and cleaning treatments apical to the point of its separation. This is significant in endodontic therapy, impacting the final treatment outcome [3]. Therefore, it is crucial to attempt instrument retrieval or bypass before considering obturation to the level of separation or resorting to surgical intervention.

The Masserann approach is unique among the many ways to remove foreign items from the root canal [4]. With success rates as high as 55%, this method is very useful for extracting intracanal silver points, broken fragments of files, and posts [5]. The armamentarium employed includes long, crown-cutting diamonds (Shofu Preparation Kit, Kyoto, Japan), Gates-Glidden drills (Mani Inc., Tochigi, Japan), a slow-speed, contra-angle handpiece (NSK, Japan), and the Masserann kit (Micro Mega, Besançon, France) [6]. By severing the surrounding radicular dentin, the Masserann kit trephine burs, which are end-cutting and color-coded, progressing in size, rotate anticlockwise to liberate space in the periphery of the separated instrument’s coronal end. The extractor resembles a tube and has a stylet or plunger rod. It seals the fragment's exposed coronal end just short of the extractor's end against an internal dent when screwed within. After that, the fragment can be eliminated by rotating anticlockwise [6]. This case report describes the successful extraction of a separated file firmly lodged in the root canal dentin of a first premolar on the right mandible.

## Case presentation

**Figure 1:**
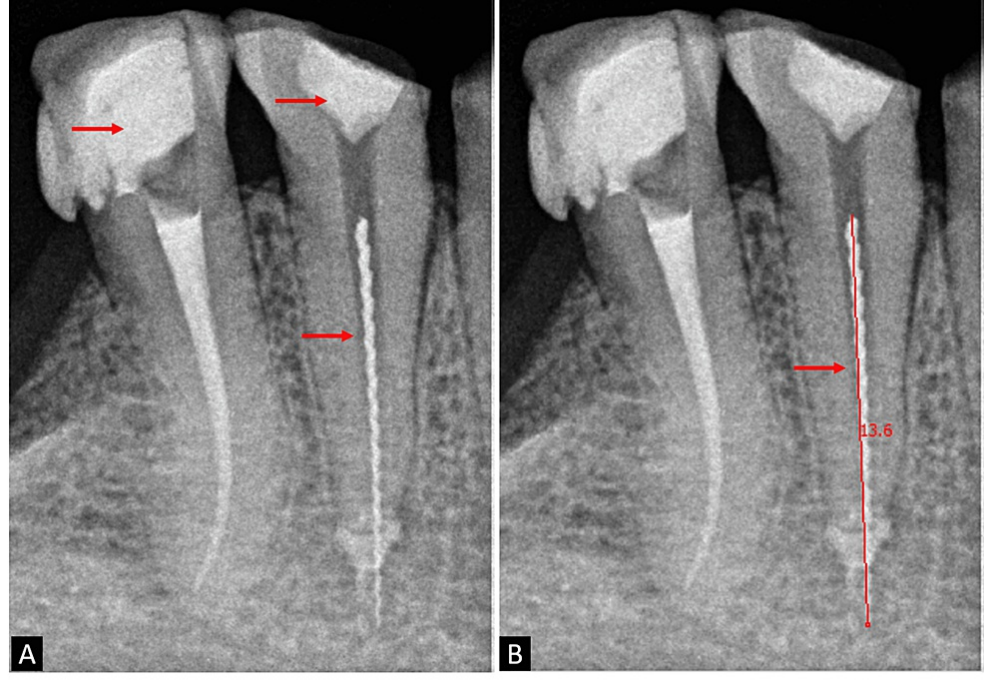
Preoperative radiograph showing 13.6 mm fractured fragment. (A) Temporary restoration seen with teeth 44 and 45, and fractured fragment seen with tooth 44. (B) A 13.6 mm fractured fragment was seen with tooth 44.

A 50-year-old male patient visited the department with a complaint of pain in the lower right back region of his jaw that had been there for three days. The pain was localized, of moderate intensity, characterized as a dull ache, and continuous. It exacerbated while lying down and during mastication. Upon clinical examination, temporary restorations were observed on teeth 44 and 45. Radiographic examination revealed a white radiopaque shadow in the root canal, indicating the presence of a separated instrument. The fragment was situated in the coronal third of the root of tooth 44, extending approximately 5 mm beyond the apex, with a length of 13.6 mm. Periapical radiolucency was evident around the separated fragment, as shown in Figure 1.

**Figure 2:**
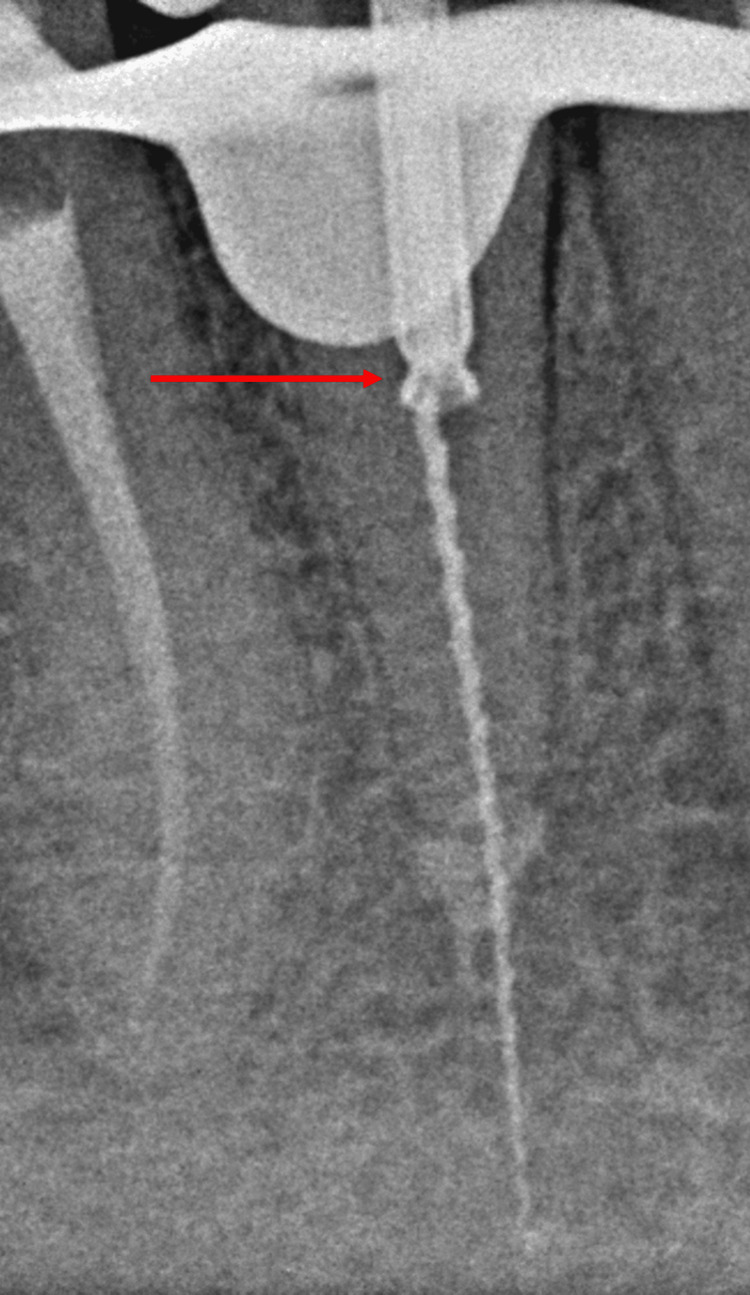
Radiograph showing the extractor tube placed over the fractured fragment.

Considering the single-rooted nature of the tooth, the absence of root canal curvature, and the fragment's location in the coronal third of the root, attempts to bypass the fragment proved unsuccessful. Therefore, the decision was made to use the Masserann technique for fragment removal. Gates-Glidden drills were used one after the other to straighten the root canal to allow radicular access to the fragment's coronal end. A contra-angle handpiece was used to attach a pre-selected 1.2 mm trephine, which was then revolved in a counter-clockwise orientation to form a trench surrounding the fragment's coronal end and remove dentin. The trephine was centered correctly over the fragment, and radiographic confirmation was obtained. The piece was then sleeved by sliding an extractor tube with a 1.2 mm diameter into the trough. The extraction tube's plunger rod was manually rotated clockwise to grasp the fragment against its wall upon radiological confirmation of its placement, as shown in Figure 2.

**Figure 3:**
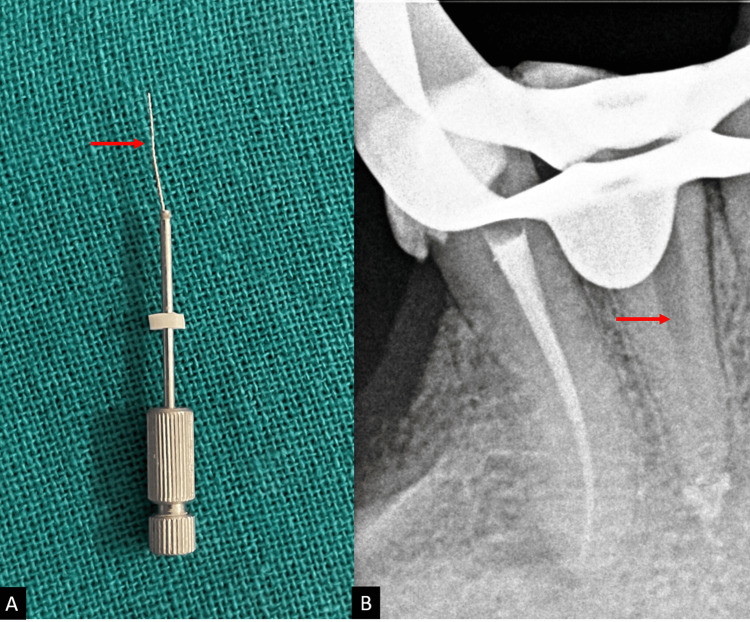
Postoperative images. (A) Image showing the extractor tube and retrieved fractured fragment. (B) Postoperative radiograph after retrieval of the fractured fragment.

The entire assemblage was spun counterclockwise to release the instrument from the dentin and enable removal as soon as the tightest hold was detected, as shown in Figure 3.

**Figure 4:**
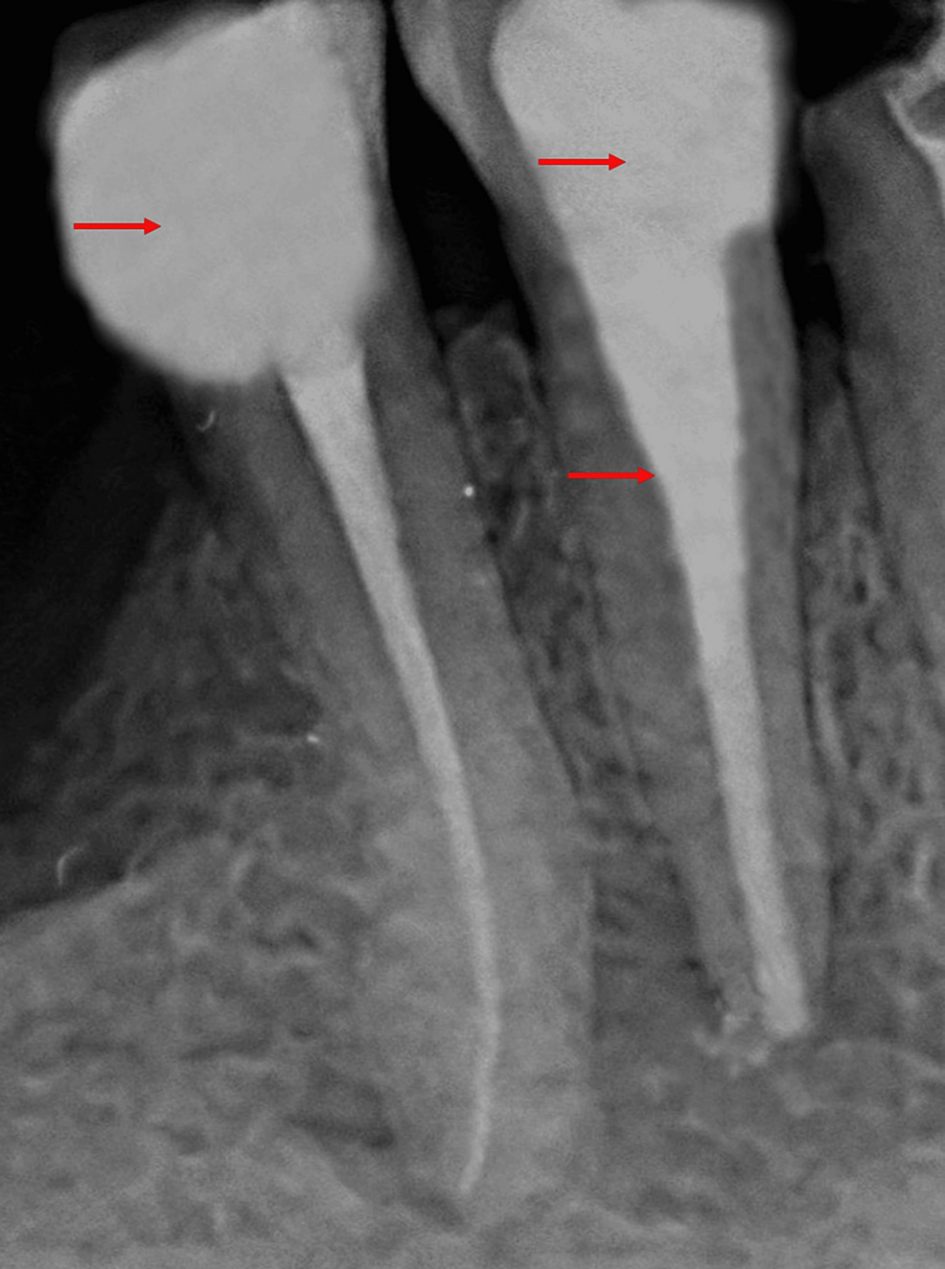
Postoperative radiograph showing obturated root canal with tooth 44 and post-endodontic restoration with teeth 44 and 45.

The working length was determined using an apex locator, with tooth 44 having a length of 14 mm. Biomechanical preparation was carried out on tooth 44 using Dentsply hand protaper files until reaching file F3. Calcium hydroxide intracanal medicament was placed, and a temporary dressing was applied to tooth 44. The patient was recalled after seven days. During the subsequent visit, the patient was asymptomatic. The temporary dressing was removed, and the canal was thoroughly irrigated with normal saline and 5.25% sodium hypochlorite. Mastercone fit was evaluated, and the canal was obturated, followed by post-endodontic restoration with teeth 44 and 45, as shown in Figure 4.

The patient was then scheduled for a follow-up after one month, during which he remained completely asymptomatic.

## Discussion

A significant obstacle to root canal cleaning and shaping is the separation of instruments within the canal, which prevents access to the apex. As a result, there is a danger to the endodontic treatment outcome and a decreased likelihood of successful retreatment [5,7]. Some of the factors that influence the prognosis in these kinds of cases are the state of the root canal (vital or nonvital), the tooth's status (symptomatic or asymptomatic, with or without periapical pathology), the level of cleaning and shaping at the time of separation, and the location of the separation within the canal. Generally speaking, the prognosis is worse than with conventional endodontic therapy [1].

As a result, every attempt should be taken to retrieve or avoid using the detached instrument. The root canal’s length, curvature, and diameter of its cross-section; thickness of dentin and root morphology; the instrument's content and cutting action (counterclockwise or clockwise); and the location, length, and degree of binding of the instrument within the canal are among the variables that affect orthograde retrieval [5].

Three instrument retrieval strategies exist, including chemical, mechanical, and surgical techniques [7]. Since surgery does not necessitate a crucial amount of dentin removal, it should be considered first when the separated fragment is mostly or entirely outside the root canal [7]. Chemical methods that corrode the fractured metallic instrument with solvents such as nitric acid, sulfuric acid, iodine trichloride, hydrochloric acid, and iodine crystals [5] or dissolve the instrument electrochemically using electrolyzed solutions of sodium chloride or fluoride [8] are inefficient for retrieving instruments because they take a significant amount of time to dissolve the metallic instrument completely. Furthermore, because these chemical solvents are limited to the shattered instrument surface in the canal, they are regarded as unpredictable and may harm the nearby soft and hard tissues [7].

There are two steps in every mechanical technique for retrieving instruments. The initial step in preparing the root canal is using ultrasonic or rotary instruments to release the broken instrument. The next step is to try to retrieve the broken instrument using ultrasonics or special equipment [7]. Mechanical methods for retrieving instruments can be broadly divided into two categories: those that use trephine bursts to penetrate the separated instrument's periphery during the preparation phase, followed by attempts to remove the instrument using equipment, and those that use ultrasonics or particular files to create a tiny space only on the fractured instrument's side during the preparation phase, followed by attempts to remove the instrument using devices or ultrasonics. Particular files and loops are included with these devices to remove the broken instruments [7]. The Canal Finder system, the EndoPuls system (EndoTechnic, San Diego, CA), and small-diameter ultrasonic tips, such as ET25 and TFRK-S, are examples of systems employing ultrasonics or specific files [7]. These devices provide a vertical movement using a handpiece and specific files, which helps bypass the separated instrument [9]. The needle-sleeve technique, Masserann kit, Endo Extractor (Brasseler Inc., Savannah, GA), Cancellier Extractor Kit (SybronEndo, Orange, CA), and Micro-Retrieve and Repair System (Superline NIC Dental, China) are mechanical devices using trephine burs in endodontic procedures. In the preparation process, these technologies uncover the coronal region of the separated instrument using a hollow tube with a cutting end and a diameter of 0.7-2.4 mm [7].

As in our case the separated instrument was present in the straight portion of the posterior teeth and most of it was present within the canal, we employed the nonsurgical mechanical method and used the Masserann kit for its retrieval. With over 30 years of experience, the Masserann kit has been used to remove damaged tools from teeth. Success rates for anterior and posterior teeth are 73% and 44%, respectively [10]. However, because using relatively large and rigid trephines can result in removing considerable quantities of root dentin, weakening the tooth, or increasing the risk of perforation, it requires regular radiographic monitoring. It may be less effective in teeth having thin or curved roots or in apically fractured fragments [3].

Notwithstanding these drawbacks, the Masserann kit works incredibly well to remove metal fillings from front teeth with robust, straight roots. The extractor's locking mechanism provides significant retention for grasping and removing firmly wedged impediments in the canal. A direct path to the fragment makes it easier for the trephine to center over it, releasing the coronal end circumferentially and safely removing the surrounding dentin. This makes it easier to grasp the fragment firmly and makes it easier to retrieve it along the root's long axis, allowing for regular retreatment [6].

Our successful attempts to remove detached files in posterior teeth using the Masserann kit challenge the literature's suggestion that employing the Masserann approach for posterior teeth can be challenging [11]. Every operation was carried out under rubber dam isolation. In one instance, the clamp's wing obscured the separated segment's appearance on the radiograph, requiring the clamp to be removed to obtain a clean image. In another instance, where the tooth was severely damaged, wedges were used to hold the rubber dam in place rather than clamps. The optimum course of action is prevention, and in situations where instruments separate, the significance of safe retrieval or bypassing is emphasized [12]. Although the Masserann procedure is time-consuming and technique-sensitive [13], detached files from maxillary lateral incisors and maxillary and mandibular molars were successfully recovered through strategic use within clinical constraints and operator expertise. On the other hand, in some situations, using ultrasonics and a dental operating microscope can increase efficacy [14].

## Conclusions

Instrument separation during endodontic therapy poses a significant challenge, affecting the success of the treatment. The presented case report demonstrates the successful removal of a tightly wedged file in a mandibular first premolar using this technique. Despite its limitations, such as the need for frequent radiographic monitoring and restricted application in certain tooth types, the Masserann kit proves effective in many cases. The importance of instrument retrieval before surgery is emphasized, underlining the technique's clinical relevance and the positive outcomes in challenging cases.
